# Organelles and phytohormones: a network of interactions in plant stress responses

**DOI:** 10.1093/jxb/erac384

**Published:** 2022-09-28

**Authors:** Andras Bittner, Agata Cieśla, Kristina Gruden, Tjaša Lukan, Sakil Mahmud, Markus Teige, Ute C Vothknecht, Bernhard Wurzinger

**Affiliations:** Plant Cell Biology, Institute of Cellular and Molecular Botany, University of Bonn, Kirschallee 1, 53115 Bonn, Germany; Laboratory of Biotechnology, Faculty of Biology, Adam Mickiewicz University, Uniwersytetu Poznańskiego 6, 61-614 Poznań, Poland; Department of Biotechnology and Systems Biology, National Institute of Biology, Večna pot 111, 1000 Ljubljana, Slovenia; Department of Biotechnology and Systems Biology, National Institute of Biology, Večna pot 111, 1000 Ljubljana, Slovenia; Plant Cell Biology, Institute of Cellular and Molecular Botany, University of Bonn, Kirschallee 1, 53115 Bonn, Germany; Department of Functional & Evolutionary Ecology, University of Vienna, Djerassiplatz 1, 1030 Vienna, Austria; Plant Cell Biology, Institute of Cellular and Molecular Botany, University of Bonn, Kirschallee 1, 53115 Bonn, Germany; Department of Functional & Evolutionary Ecology, University of Vienna, Djerassiplatz 1, 1030 Vienna, Austria

**Keywords:** Abscisic acid (ABA), chloroplast, ethylene, jasmonates, mitochondria, plant organelles, phytohormones, plastids, salicylic acid (SA), retrograde signaling, stress signaling

## Abstract

Phytohormones are major signaling components that contribute to nearly all aspects of plant life. They constitute an interconnected communication network to fine-tune growth and development in response to the ever-changing environment. To this end, they have to coordinate with other signaling components, such as reactive oxygen species and calcium signals. On the one hand, the two endosymbiotic organelles, plastids and mitochondria, control various aspects of phytohormone signaling and harbor important steps of hormone precursor biosynthesis. On the other hand, phytohormones have feedback actions on organellar functions. In addition, organelles and phytohormones often act in parallel in a coordinated matter to regulate cellular functions. Therefore, linking organelle functions with increasing knowledge of phytohormone biosynthesis, perception, and signaling will reveal new aspects of plant stress tolerance. In this review, we highlight recent work on organelle–phytohormone interactions focusing on the major stress-related hormones abscisic acid, jasmonates, salicylic acid, and ethylene.

## Teaser

A review of organelle–phytohormone interactions and retrograde signals, highlighting recent work and open questions and focusing on the stress-related hormones abscisic acid, jasmonates, salicylic acid, and ethylene.

## Introduction

Organelles have essential functions in most cellular processes including growth and development of plants. As a consequence of their endosymbiotic origin, mitochondria and chloroplasts carry their own genomes. However, the majority of organellar proteins are now nucleus encoded (Zimorski *et al.*, 2014). Development and plant fitness require the coordination of organellar functions, including coordinated expression of genes encoded in the three genomes of the plant cell. ­Therefore, organelles constantly transmit their physiological state as intracellular signals to the nucleus for the coordination of gene expression. The discovery of stress-related organellar signals led to the assumption that plant organelles are one of the primary sites for sensing environmental changes (reviewed in Kleine and Leister, 2016; Kmiecik *et al.*, 2016; Crawford *et al.*, 2018; Dopp *et al.*, 2021; Li and Kim, 2022). Work has revealed different stress-related organelle signaling components such as the carotenoid oxidation byproducts (Ramel *et al.*, 2012), phosphoadenosine 5ʹ-phosphate (PAP) (Estavillo *et al.*, 2011), executer-mediated response to singlet oxygen (Lee Keun *et al.*, 2007), and the isoprenoid-precursor methylerythritol cyclodiphosphate (Xiao *et al.*, 2012).

**Fig. 1.**
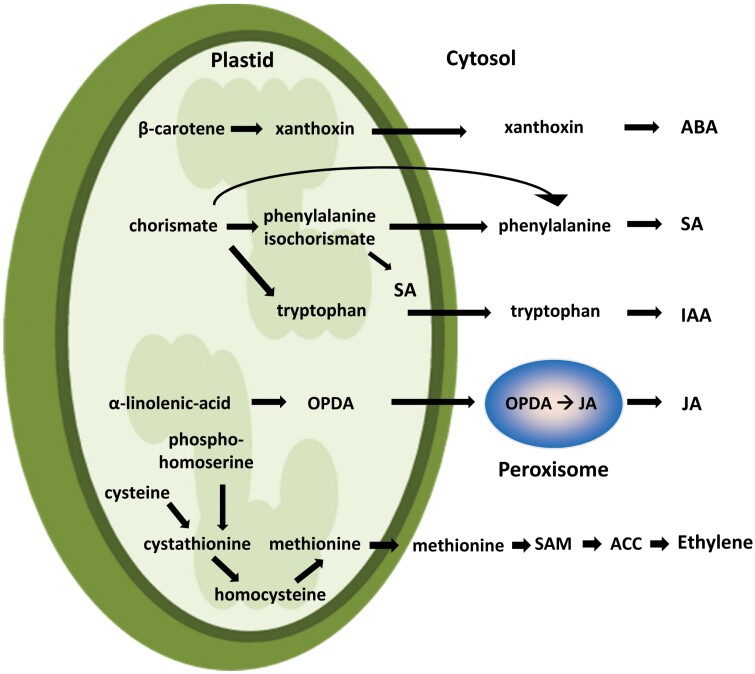
The chloroplast as metabolic hub for phytohormone precursors. Numerous phytohormone pathways start with secondary metabolism in plastids: xanthoxine biosynthesis via the xanthophyll cycle—the precursor of ABA; chorismate biosynthesis—the precursor of SA and auxin (IAA); oxidized lipids such as linolenic acid—the precursor of jasmonates (via JA); cystathionine—the precursor for methionine and thus ethylene. ACC: 1-aminocyclopropane 1-carboxylate; IAA, indole-3-acetic acid; OPDA, oxophytodienoic acid; SAM, *S*-adenosyl-l-methionine.

In addition to their role as environmental sensors, organelles (especially chloroplasts) are the main hub for the metabolism of several phytohormone precursors, as illustrated in Fig. 1 (for a recent review see Fàbregas and Fernie, 2021). Phytohormones are associated with various physiological and metabolic processes (Cackett *et al.*, 2022), and it is becoming clear that they constitute a network that is interconnected at multiple levels. Antagonistic and synergistic interactions between different hormones have been described during biotic (Berens *et al.*, 2017; Aerts *et al.*, 2021) and abiotic stress. For example, synergistic effects of jasmonic acid (JA) with salicylic acid (SA) were found in the high-light response (D’Alessandro *et al.*, 2020), or between abscisic acid (ABA) and JA in the drought-stress response (Liu *et al.*, 2016), while antagonistic effects of ABA and cytokinins have been reported under drought stress (Huang *et al.*, 2018). Finally, ethylene was shown to modulate the activity of SA in the pathogen response (Ramšak *et al.*, 2018).

**Fig. 2.**
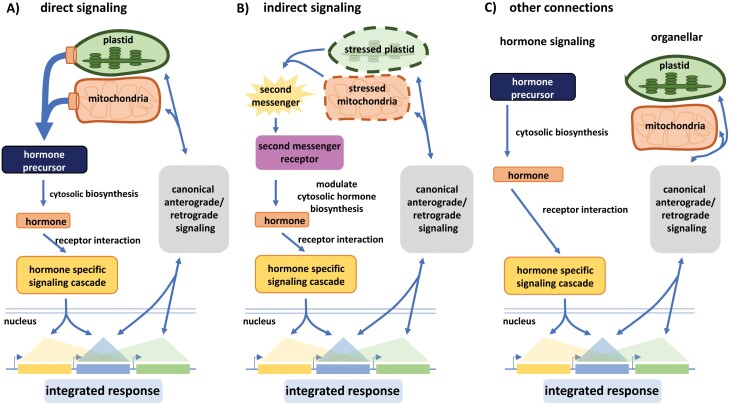
Direct/indirect signals in regulation of gene expression. Different types of connections between organelles and hormone signaling affect gene expression. (A) Direct signals: hormone levels, depending on precursor metabolites for hormone biosynthesis of exclusive organellar origin, regulate a subset of genes (yellow) while another subset of genes is controlled by retrograde signals from organelles (green), and a third subset of genes is regulated by both input pathways (blue). (B) Indirect signals: hormone levels regulating a subset of genes are indirectly altered via second messenger molecules of organellar origin, modulating the enzymes relevant for hormone biosynthesis. (C) Other signaling connections: transcriptional changes that do not fall into (A, B) but require a functional hormonal signaling cascade and a functional organellar retrograde signaling cascade at the same time.

Classically, hormone signaling is discussed in a historical context where specific hormones are often viewed in a particular developmental or stress context. For this review, however, it seemed more reasonable for us to structure organelle–hormone signals to cover (i) direct signaling connections, (ii) indirect signals, and (iii) other, more complex signaling interactions (Fig. 2). Direct signals include metabolites of exclusive organellar origin that serve as key precursors for the final steps of plant hormone biosynthesis. The active forms of the hormones present the source of the signal, triggering a receptor-based signaling cascade leading to an integrated transcriptional response. The regulated genes could be exclusively under the control of a certain hormonal signaling cascade, controlled by retrograde signals from organelles, or controlled by both input pathways. Alternatively, in an indirect mechanism, the second messenger metabolites of organellar origin are perceived by receptors elsewhere in the cytoplasm and thereby act as signals leading to a modulation of biosynthetic activities of enzymes involved in hormone biosynthesis. The elicitation of organellar second messengers may be triggered via anterograde signals. Similar to the direct signaling pathways, the resulting integrated transcriptional response is a combination of exclusively hormone-derived transcriptional changes, transcriptional changes elicited by organellar retrograde signals, and a combination of both. Finally, transcriptional changes may require a functional hormonal signaling cascade and a functional organellar retrograde signaling cascade at the same time to provide an integrated transcriptional response in order to react to an environmental change, thus illustrating more complex signaling interactions (Fig. 2).

In this review, we summarize recent work on organelle–phytohormone interactions to highlight and address open questions within these interwoven signaling cascades, concentrating on the stress-related hormones ABA, jasmonates, SA, and ethylene. However, other phytohormones also have tight relations to plant organelles, and the impact of auxin, cytokinins, gibberellins, and strigolactones on photosynthesis and photoprotection was covered in a recent review by Müller and Munné-Bosch (2021).

## Abscisic acid

ABA was identified in the 1960s as an endogenous signal (Ohkuma *et al.*, 1963). Since then, numerous functions of ABA in plant development and adaptation have been described, including reprogramming of gene expression, protection of photosynthesis, stomatal closure, maturation of seeds, and primary root growth (for recent reviews on ABA see Ma *et al.*, 2018; Seo and Marion-Poll, 2019; Cardoso *et al.*, 2020). Besides these diverse functions, meta-analysis of microarray studies under different operational retrograde signaling conditions, such as transition from low light to high light intensities, abolishment of the tetrapyrrole pathway by gabaculine treatment, or manipulation of electron flow via mutation of the Ser/Thr protein kinase STN7, identified ABA as a major component within the core network of inter-organelle signaling of Arabidopsis (Gläßer *et al.*, 2014). Many nuclear genes that encode organellar proteins related to photosynthesis carry ABA responsive elements in their promoter (Koussevitzky *et al.*, 2007), and exogenously applied ABA represses the transcription of almost all genes encoded by the plastome in Arabidopsis and barley (Yamburenko *et al.*, 2013; Yamburenko *et al.*, 2015). Therefore, it is no surprise that ABA is important in coordinating the response of the organelle that produces the intermediates for its own biosynthesis (the chloroplast) and that ABA signaling is linked with inter-organelle communication via direct and indirect connections.

### Direct connection of chloroplast functions and ABA biosynthesis

**Fig. 3.**
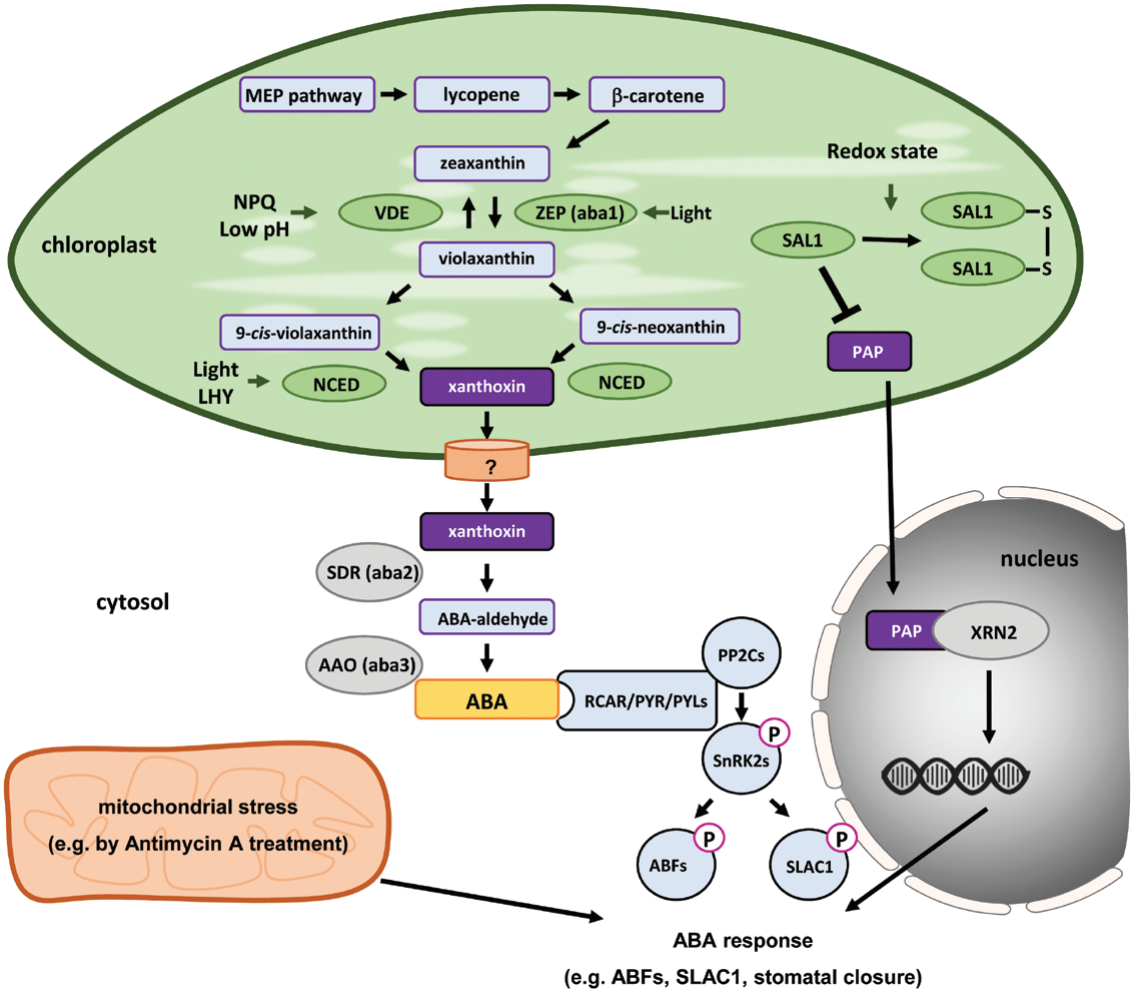
The role of organelles in abscisic acid (ABA) signaling. ABA biosynthesis is initiated in chloroplasts by the non-mevalonate (MEP^−^) pathway and continuous via phytoene, lycopene, β-carotene, and zeaxanthin biosynthesis. The xanthophyll cycle converts zeaxanthin into violaxanthin, which is converted into xanthoxin. In the cytosol, xanthoxin is the substrate for the synthesis of ABA aldehyde and finally ABA. ABA is perceived through receptors from the RCAR/PYR/PYL family and PP2C co-receptors, which in turn activate SnRK2s and ABFs by phosphorylation. Chloroplast signals and functions, such as the SAL–PAP pathway and NPQ, control ABA biosynthesis and signaling. AAO, ABA-aldehyde oxidase; ABF, ABA responsive element-binding factor; LHY, late hypocotyl elongation factor; NCED, 9-*cis*-epoxycarotenoid dioxygenase; NPQ, non-photochemical quenching; PAP, 3ʹ-phosphoadenosine-5ʹ-phosphate; PP2C, protein phosphatase 2C; SAL1, dinucleotide phosphatase/inositol phosphate phosphatase; SDR, short chain dehydrogenase; SLAC1, slow anion channel-associated 1; SnRK2s, SNF1-related protein kinases; VDE, violaxanthin de-epoxidase; XRN, exoribonucleases; ZEP, zeaxanthin epoxidase.

The biosynthetic pathway of ABA is closely associated with plastid functions and starts with the hydroxylation of β-carotene to zeaxanthin, followed by subsequent conversion to violaxanthin through the xanthophyll cycle (Fig. 3; for details see Seo and Marion-Poll, 2019). Violaxanthin is then cleaved by 9-*cis*-epoxycarotenoid dioxygenase (NCED), which leads to xanthoxin. The oxidative cleavage via NCED is the first irreversible conversion step towards ABA and well accepted as a bottleneck in ABA biosynthesis (Endo *et al.*, 2008). Finally, xanthoxin is transferred to the cytosol by an unknown process, where it is converted first to abscisic aldehyde and then to ABA. The ABA precursors xanthoxin and abscisic aldehyde can be found in numerous plant tissues, but reciprocal grafting of ABA biosynthetic mutants with wild type plants showed that leaves are the predominant site for the final steps of ABA biosynthesis (McAdam *et al.*, 2016).

Several components of the ABA biosynthesis pathway are directly connected to chloroplast function and signaling. For instance, one of the most rapid components of the photo-oxidative stress response is decreased pH in the thylakoid lumen via non-photochemical quenching to rebalance photosynthesis (Ruban *et al.*, 1992). The low pH affects the xanthophyll cycle by activating violaxanthin de-epoxidase, which converts violaxanthin into zeaxanthin and thereby counteracts the first steps of ABA biosynthesis (Pastori *et al.*, 2003).

### Indirect connection of chloroplast and ABA signaling

Besides the direct control of ABA precursor availability by chloroplast functions, ABA biosynthesis is also under the indirect control by pathways that are modulated by organelles. One example is the impact of the circadian clock, which is under the control of chloroplast functions. Genome-wide binding analysis in Arabidopsis revealed that several genes involved in ABA biosynthesis are controlled by the LATE HYPOCOTYL ELONGATION FACTOR (LHY), an important component of the molecular clock in plants (Adams *et al.*, 2018). Subsequent analysis with *LHY* overexpression and loss of function lines revealed that LHY negatively effects transcription of *NCED3*, the gene for the most abundant NCED in Arabidopsis (Adams *et al.*, 2018). In addition, *LHY* overexpression represses the accumulation of ABA during drought. Perturbation of chloroplast-related functions by chemicals such as 3-(3,4-dichlorophenyl)-1,1-dimethylurea, paraquat, and ascorbate modifies the pace of nuclear-driven circadian oscillations (Philippou *et al.*, 2020), which led to the hypothesis that the circadian rhythm could act as an additional feedback mechanism from chloroplasts on ABA biosynthesis, due to connection of chloroplast functions and the circadian clock.

In addition to ABA biosynthesis, ABA signaling is widely interconnected with retrograde signals from chloroplasts. For instance, PAP signaling supports the ABA response pathway during stomatal closure and seed maturation via activation of the slow anion channel, SLAC1 (Pornsiriwong *et al.*, 2017). PAP is a 3ʹ-phosphorylated-nucleotide that is maintained at very low levels by the action of the phosphatase SAL1 (also called ALX8, FIERY1, and HOS2). During photo-oxidative stress, SAL1 is inactivated by oxidation, which in turn leads to an accumulation of PAP and triggers several chloroplast stress signals, such as high expression of the H_2_O_2_ scavenger ascorbate peroxidase 2 under excess light (Estavillo *et al.*, 2011). ABA is known as a major player in stomatal closure, as several ABA-insensitive mutants showed abolished stomatal closure under drought conditions (Chater *et al.*, 2015). Pornsiriwong *et al.* (2017) showed that SAL1 inactivation/PAP accumulation can restore the guard cell responsiveness in the ABA-insensitive mutant lines *ost1-*2 and *abi1-1* via interaction with the ABA signaling pathway downstream of these genes by the phosphorylation and activation of SLAC1.

Promoter and transcript analysis of *2-Cys PEROXIREDOXIN A* (*2CPA*), encoding a highly abundant reactive oxygen species (ROS) scavenger in chloroplasts, revealed that its expression is controlled by ABA together with the cellular redox state (Baier *et al.*, 2004). External application of ABA decreased the expression of *2CPA* and consequently several ABA-insensitive mutants showed an increased expression of *2CPA* (Baier *et al.*, 2004). To what extent ABA-controlled expression of *2CPA* could potentially feed back on the redox-responsive SAL1 inactivation/PAP accumulation is an open question.

### Complex interactions: ABA signaling, plastid development, and nuclear interaction

Besides the direct connection of chloroplast functions with ABA biosynthesis and the indirect connection of ABA with operational retrograde signals, ABA itself can also shape plastid development and properties, which can be hypothesized as being an additional, more complex layer of ABA control within retrograde and anterograde signaling.

Transient expression of redox biosensors in the cytosol, the chloroplast stroma, and the nucleus revealed that photosynthesis-derived H_2_O_2_ in the chloroplast directly alters the H_2_O_2_ content of the nucleus, without affecting the cytosolic redox pool (Exposito-Rodriguez *et al.*, 2017). It is proposed that the transfer of H_2_O_2_ occurs as a retrograde signal via the tubular structures of the chloroplast known as stromules. Gray *et al.* (2012) showed that ABA triggers the formation of chloroplast–nucleus complexes via stromules in tobacco and wheat seedlings. Vice versa, treatment with inhibitors of ABA biosynthesis prevents stromule formation under stress conditions. Recent work with strigolactone mutant lines and strigolactone biosynthesis inhibitors furthermore showed that ABA-induced stromule formation depends on active strigolactone biosynthesis in plastids, indicating crosstalk between ABA and strigolactones in this process (Vismans *et al.*, 2016).

Besides signals from mature chloroplasts in response to environmental constraints (referred to as operational retrograde signals), retrograde signaling pathways also act during chloroplast development to control transcription of nucleus-encoded genes related to photosynthesis (referred to as biogenic retrograde signals). These biogenic signals are mainly studied by inhibition of carotenoid biosynthesis with norflourazon and by inhibition of the translation machinery in plastids with lincomycin (Susek *et al.*, 1993). Screens for mutant lines that are insensitive to norflourazon and lincomycin revealed six gene loci, called *GENOME UNCOUPLED 1–6* (*GUN1–6*). The majority of photosynthesis-related nucleus-encoded genes are repressed by ABA treatments (Yamburenko *et al.*, 2015). Due to a certain overlap of misregulated genes between *gun1-1* and *abi4* knockout plants and suppression of the *gun1-1* phenotype in *ABA INSENSITIVE 4* (*ABI4*) overexpression plants, it was proposed that ABA signaling via the transcription factor ABI4 is an important downstream component of GUN1-mediated chloroplast biogenesis (Koussevitzky *et al.*, 2007). However, recent work has re-evaluated the role of the ABA signaling component ABI4 in chloroplast biogenesis. Analysis of the phenotype of four different *ABI4* alleles (*abi4-102*, *abi4-1*, *abi4-2*, and *abi4-4*) questions a role of ABI4 in GUN1-mediated biogenic control of photosynthesis-related, nucleus-encoded genes (Kacprzak *et al.*, 2019). On the other hand, Zhu *et al.* (2020) showed recently that *ABI5* overexpression suppresses chloroplast development and expression of photosynthesis-related genes in potato (*Solanum tuberosum*). Overall, the exact way that ABA is integrated in the biogenic control of chloroplast morphogenesis is still an open question.

### ABA signaling and mitochondrial stress response

Inhibition of mitochondrial electron transport, for example by the chemical antimycin A, can trigger the transcription of ­mitochondrial stress-responsive genes. The best-known example is the increased transcript abundance of the nucleus-encoded *ALTERNATIVE OXIDASE 1A* (*AOX1a*) after perturbation of mitochondrial electron transport (Clifton *et al.*, 2006). A forward genetic screen identified several *REGULATORS OF AOX1A* (*RAOs*) genes and thereby components of mitochondrial retrograde signaling. One of the first identified RAOs is the DREB transcription factor ABI4 (Giraud *et al.*, 2009). Application of ABA leads to suppression of *ABI4* expression and by that releases the repression of *AOX1a*, suggesting a positive role of ABA in mitochondrial retrograde signaling (Yao *et al.*, 2015). It has also been reported that the induction of *ABI4* and *AOX1A* transcription depends on RETARDED-ROOT GROWTH-LIKE FACTOR 1 (RRL1), a mitochondria-localized protein involved in mitochondrial retrograde signaling (Yao *et al.*, 2015). Nevertheless, the full impact of ABA on mitochondrial function is still under investigation.

## Jasmonates

### Direct connection of organellar functions and jasmonate biosynthesis

**Fig. 4.**
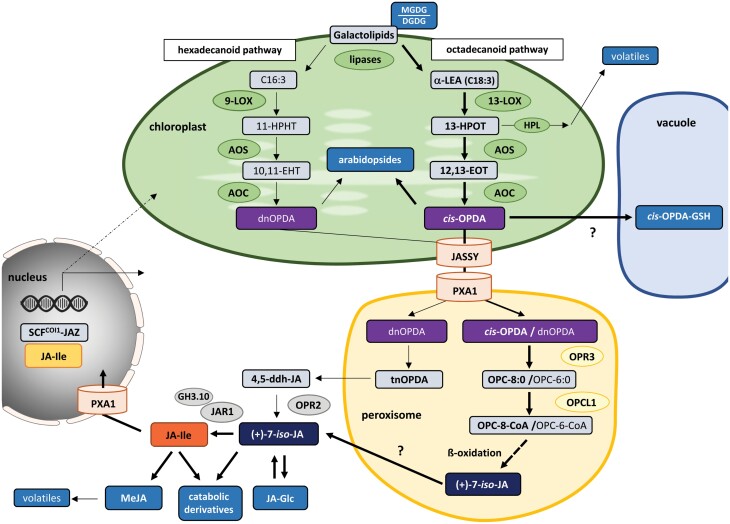
The role of organelles in jasmonate signaling. The term jasmonate comprises JA and JA-Ile as well as several precursors and catabolic derivatives some of which also possess bioactivity. Jasmonate biosynthesis is initiated in chloroplasts by oxidation of C18:3 (octadecanoid pathway) and C16:3 (hexadecanoid pathway) fatty acids derived from galactolipids, which are converted in several steps to the first committed precursor, OPDA. The MDGD/DGDG ratio, but also conjugation of OPDA to GSH or esterification to galactolipids (arabidopsides), affects OPDA homeostasis. Biosynthesis of JA continues in peroxisomes by β-oxidation of OPC-8:0 and OPC-6:0. A minor, less well described bypass pathway of JA formation involves tnOPDA and 4,5-ddh-JA. JA-Ile, the most bioactive of the jasmonates, is finally synthesized in the cytosol from JA and isoleucine, the latter being derived from methionine also made in chloroplasts. Ultimately, JA-Ile exerts its action in the nucleus by promoting the formation of SCF^COI1^–JAZ co-receptor complexes and thus releasing JAZ-dependent gene suppression. 4,5ddh-JA, 4,5-didehydro-jasmonate; 10,11-EHT, 10,11(*S*)-epoxy-hexadecatrienoic acid; 11-HPHT, 11(*S*)-hydroperoxy-hexadecatrienoic acid; 12,13-EOT, 12,13(*S*)-epoxy-octadecatrienoic acid; 13-HPOT, 13(*S*)-hydroperoxylinolenic acid; α-LEA, α-linolenic acid; AOC, allene oxide cyclase; AOS, allene oxide synthase; DGDG, digalactosyl-diacylglycerol; GH3.10, glycoside hydrolase 3 gene family 10; HPL, hydroperoxide lyase; JA, jasmonic acid; JA-Glc, glycosylated jasmonate; JAR1, jasmonate-resistant 1; JASSY, chloroplast jasmonate transporter; LOX, lipoxygenases; MeJA, methyl jasmonate; MGDG, monogalactosyldiacylglycerol; OPC, 3-oxo-2-(2^0^-[*Z*]-pentenyl)-cyclopentane-1-octanoic acid; OPCL1, OPC-8:0 CoA ligase 1; OPDA, oxophytodienoic acid; OPR, OPDA reductase; PXA1, peroxisomal ABC-transporter 1.

Jasmonate is a collective term used for jasmonic acid (JA) and a diverse set of precursors and derivatives. They regulate a plethora of processes ranging from defense against stresses to the regulation of plant growth and development (reviewed in Wasternack and Hause, 2013; Koo, 2018). The first committed precursors of JA, 12-oxophytodienoic acid (*cis*-OPDA) and dinor-12-oxo-10,15(*Z*)-phytodienoic acid (dnOPDA), are made in chloroplasts (Fig. 4). As they are oxylipin derivatives, their biosynthesis starts with free fatty acids released from membrane lipids by the action of various lipases. In the so-called octadecanoid pathway, the initial substrate of *cis*-OPDA is α-linolenic acid (C18:3) released from the chloroplast galactolipids monogalactosyldiacylglycerol (MGDG) and digalactosyldiacylglycerol (DGDG). By contrast, dnOPDA is synthesized via a parallel hexadecanoid pathway from the less abundant 16:3 fatty acid roughanic acid (Bannenberg *et al.*, 2009). Jasmonate synthesis seems to be closely interconnected with the MDGD/DGDG ratio since a mutant defective in DGDG production displays an increased production of *cis*-OPDA, as well as JA and jasmonoyl isoleucine (JA-Ile) (Lin *et al.*, 2016).

Both OPDA variants can be found esterified to galactolipids (reviewed in Genva *et al.*, 2019). First identified in Arabidopsis and called arabidopsides, they have now also been detected in other plants. Due to their rapid increase upon certain biotic stresses, a role in plant defense was suggested. Oxylipins also have been found as glutathione (GSH) conjugates whose amount increases upon pathogen attack (Davoine *et al.*, 2005). *cis*-OPDA–GSH conjugates are transported into the vacuole for either sequestration or degradation (Ohkama-Ohtsu *et al.*, 2011). While the exact function of *cis*-OPDA–GSH and the arabidopsides remains elusive, they could be involved in the removal of a stimulus-induced excess of OPDA, or vice versa be an additional source for rapid jasmonate formation (Böttcher and Weiler, 2007).

Formation of JA from *cis*- and dnOPDA is continued in the peroxisome and the cytosol by two different routes (Fig. 4). Conjugation of JA to Ile by JASMONATE-RESISTANT 1 (JAR1), and to a lesser extent by other GH3 family proteins such as GH3.10, generates JA-Ile (Staswick and Tiryaki, 2004; Delfin *et al.*, 2022). Isoleucine is derived from threonine and methionine made in chloroplasts. Thus, JA-Ile formation is independently connected twice to chloroplast metabolism. Most studies suggest that JA-Ile is the major biologically active JA derivative because it can promote the formation of SCF–COI1–JAZ co-receptor complexes involved in jasmonate-mediated transcriptional regulation (reviewed in Koo, 2018).

JA and JA-Ile are both substrates for further derivatization. Although these compounds are mainly considered as catabolic intermediates, bioactivity has nevertheless been suggested for some of them (reviewed in Koo, 2018; Wasternack and Hause, 2013). A notable derivative is methyl jasmonate (MeJA), a volatile communication molecule, present for example in essential oils of the jasmine flowers, where it was the first jasmonate ever identified (Demole and Stoll, 1962). After uptake into the cell, MeJA is converted to JA (Tamogami *et al.*, 2008), thereby facilitating the external induction of jasmonate signaling. Interestingly, an intermediate of *cis*-OPDA synthesis, 13(*S*)-hydroperoxylinenic acid (13-HPOT), also gives rise to various volatiles important in the odors of fruits and vegetables (Matsui, 2006). It is not known, however, how the formation of these volatiles is interconnected with jasmonate biosynthesis and/or jasmonate signaling.

Jasmonate-mediated transcriptional changes are known to affect a wide range of cellular processes including organellar functions and expression of nucleus-encoded organellar proteins. However, comparatively little is known about organellar processes directly regulated by jasmonate signaling. Several studies showed differential effects of external jasmonate application, *JAR1* overexpression, or jasmonate signaling suppression on the expression of nucleus-encoded photosynthesis-related genes (Attaran *et al.*, 2014; Sirhindi *et al.*, 2020; Mahmud *et al.*, 2022). Recent findings on Arabidopsis also showed enhanced expression of most of the chloroplast-encoded genes by JA treatment (Zander *et al.*, 2020) or *JAR1* overexpression (Mahmud *et al.*, 2022). It remains unclear whether this is a direct effect on the transcription of these genes within the organelle.

The effect of jasmonate on chlorophyll degradation has been known for a very long time (Ueda and Kato, 1980). Several studies have since reported that JA signaling influences leaf senescence by induction of SENESCENCE-ASSOCIATED GENES (SAGs) and CHLOROPHYLL CATABOLIC GENES (CCGs) involved in chlorophyll breakdown (Shan *et al.*, 2011; Zhu *et al.*, 2015). Regulation occurs via increase in the expression of JA-Ile-dependent MYC type ­transcription factors, which in turn modulate the expression of genes that facilitate chlorophyll breakdown (Zhu *et al.*, 2015). Regulation also occurs more indirectly by MYC-mediated increase in expression of some NAC type transcription factors that were found to regulate leaf senescence. Indeed, a partially overlapping set of genes seems to be regulated by MYC2/3/4 and NAC019/055/072. By contrast, expression of *RUBISCO ACTIVASE* (*RCA*) was down-regulated by jasmonate in a COI1-dependent manner (Shan *et al.*, 2011). Since the *rca1* mutant displays typical senescence-related features and several senescence-promoting genes show up-regulation in the mutant, RCA could play an important role in jasmonate-induced de-greening (Shan *et al.*, 2011).

### Indirect mechanisms of jasmonate function

Jasmonate signaling is best described for the direct effect of JA-Ile on gene transcription via the SCF–COI1–JAZ co-receptor complex. Very little is known about indirect jasmonate signaling or other functions related to chloroplasts or mitochondria as depicted in Fig. 2B, C.

Besides JA-Ile, the most bioactive jasmonate, biological functions have been suggested for the chloroplast-derived precursor OPDA (recently reviewed in Maynard *et al.*, 2018; Liu and Park, 2021). OPDA is found in marine algae, terrestrial algae, mosses, and ferns, but the full jasmonate synthesis pathway is absent in these organisms. Recent work suggested that dnOPDA is the evolutionary precursor of JA-Ile and can interact with the COI receptor orthologs of mosses (Monte *et al.* 2018; Inagaki *et al.*, 2021). These findings indicate that the origin of jasmonate signaling can be directly connected to the chloroplast.

OPDA signaling seems to act both independently of and in concert with JA-Ile and it moreover affects the transcription of both COI1-dependent and -independent genes (Taki *et al.*, 2005; Stinzi *et al.*, 2001). Indeed, a signaling function of OPDA independent of JA was already suggested in the 1990s (reviewed in Bӧttcher and Pollmann, 2009). Many aspects of this OPDA-mediated signaling are not well understood but there is evidence for an action by binding to proteins such as 13-lipoxygenase, cyclophilin 20-3, thioredoxin (TRX)-m4, and peroxiredoxin (Dückershoff *et al.*, 2008). Park *et al.* (2013) subsequently described that reversible binding of OPDA to cyclophilin 20-3 promotes the formation of a complex between cysteine synthase and *O*-acetylserine (thiol) lyase B. This leads to an increase in thiol metabolites and the cellular redox potential (Park *et al.*, 2013). This redox change then plays a role in the expression of OPDA-dependent genes. By contrast, OPDAylation describes the covalent binding of OPDA to protein thiols (Maynard *et al.*, 2021). Such thiols are targets of multiple post-translational modifications as part of what is called the thiol switch, and OPDAylation could be another player in this regulatory system. Recently, Knieper *et al.* (2022) analysed the *in vitro* interaction of several redox transmitters with OPDA under physiological OPDA concentrations. They obtained evidence for OPDAylation of both the cytosolic TRX-h3 and TRX-h5 and the chloroplastic TRX-f1 and TRX-m4. The *in vivo* relevance of this finding needs to be further analysed, but overall these works substantiate the signaling role of OPDA and suggest a close connection to redox-mediated processes.

Extracellular ATP (eATP) acts as a ‘damage-associated molecular pattern’ signal and is associated with a number of secondary messengers. Wounding, which induces ATP release, also induces expression of jasmonate-dependent genes. Tripathi *et al.* (2018) recently demonstrated that eATP activates these genes not via jasmonate biosynthesis but by direct enhancement of COI1–JAZ1 interaction followed by JAZ1 protein degradation. Moreover, this effect likely involves the secondary messengers Ca^2+^, NO, and ROS induced by eATP (Tripathi *et al.*, 2018).

Proteomic studies suggested that the effect of MeJA application on root growth includes a reduction of proteins involved in ATP synthesis, thus affecting mitochondrial energy metabolism (Cho *et al.*, 2007; Ruiz-May *et al.*, 2011). Early on it was suggested that jasmonate signaling induces H_2_O_2_ production upon herbivory (Orozco-Cárdenas *et al.*, 2001), and similarly jasmonate-induced ROS production in mitochondria was suggested to play a role in defense-related programmed cell death (Zhang and Xing, 2008). Also, MeJA-induced inhibition of root growth at least in hairy-root cultures is believed to involve cell death. Loyola-Vargas *et al.* (2012) found that treatment of hairy roots with MeJA enhances mitochondrial H_2_O_2_ accumulation together with an increased activity of corresponding anti-oxidant enzymes, and concluded that jasmonate signaling induced the oxidative burst that subsequently alters the mitochondrial proteome and mitochondrial function.

Mechanisms not directly related to jasmonate-mediated transcriptional regulation also seem to be involved in the initiation of jasmonate biosynthesis. Recently, Kimberlin *et al.* (2022) suggested that post-transcriptional processes, such as protein stability, play a role in the regulation of lipases that initiate the fast release of α-linolenic acid from chloroplast lipids upon wounding. Indeed, they suggest that positive feedforward mechanisms, i.e. jasmonate-mediated transcriptional activation of jasmonate biosynthesis pathway enzymes, have a fine-tuning role and exclude not only the lipases but also JAR1. However, these non-transcriptional regulations involved in regulating OPDA formation are not well understood.

## Ethylene

Ethylene (IUPAC name: ethene) plays a central regulatory role throughout the whole plant life cycle from germination to integration of environmental changes to fruit ripening. Given its importance, the role of ethylene in plant growth, its biosynthesis pathways, and its downstream signaling pathways have been under constant investigation since its discovery as a plant growth factor by Neljubow (1901). That research led to a wealth of knowledge in plant ethylene signaling, which is reviewed in recent publications (Bakshi *et al.*, 2015; Binder, 2020; Pattyn *et al.*, 2021). Currently, ethylene is increasingly recognized also as an important mediator of stress signaling, with connections to plastid as well as mitochondrial signaling.

### Direct signaling connections

**Fig. 5.**
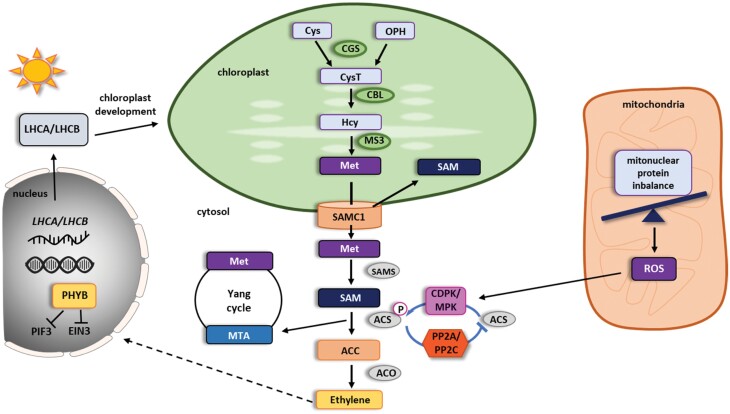
The role of organelles in ethylene signaling. The chloroplast-localized enzyme cystathionine γ-synthase (CGS) catalyses the formation of cystathionine (CysT) from cysteine (Cys) and *O*-phosphohomoserine (OPH). Cystathionine is further transformed into homocysteine (Hcy) by cystathionine β-lyase (CBL). In the next step, the chloroplast-localized isoform of the methionine synthase 3 (MS3) forms methionine (Met), which in turn is transported out of the chloroplasts by SAMC1. In the cytoplasm Met is directly transformed into *S*-adenosylmethionine (SAM) by *S*-adenosylmethionine synthase (SAMS). SAM as main methyl donor is transported also back to the chloroplast by SAMC1. In the cytoplasm, SAM is converted into 1-aminocyclopropane 1-carboxylate (ACC) by the rate limiting ACC synthases (ACSs). After phosphorylation by calcium-dependent kinases (CDPK) and/or MAP kinases (MPK), ACSs are stabilized and therefore activated. Dephosphorylation of ACSs by different protein phosphatases (PP2A, PP2C) destabilizes the protein and leads to an immediate loss of ACS activity. The by-product of the reaction conducted by ACS, 5ʹ-methylthioadenosine (MTA), enters the Yang cycle to be detoxified and recycled into Met. ACC oxidase (ACO) catalyses the final step from ACC to ethylene. The mitonuclear protein imbalance leads to increase of mitochondrial reactive oxygen species (ROS) level. As a consequence, the mitochondrial unfolded protein response (UPRmt) is initiated. The elevated ROS level activates MPK6, which in turn promotes ET production by two ways: by phosphorylation of ACS6 and increase in transcription of the *ACS6* gene. Additionally, nuclear ETHYLENE INSENSITIVE 3 (EIN3), a major ethylene responsive transcription factor, also plays a part in anterograde signaling (dashed line) in the chloroplast. After dark to light transition, PHYB promotes EIN3 and PIF3 degradation leading to LHCA and LHCB expression and chloroplast development.

Ethylene biosynthesis is usually described as a linear two step reaction in the cytosol where aminocyclopropane 1-carboxylate synthase (ACS) converts *S*-adenosyl-l-methionine (SAM) to 1-aminocyclopropane 1-carboxylate (ACC) and 5ʹ-methylthioadenosine (MTA) (Adams and Yang, 1979; Boller *et al.*, 1979). Subsequently, 1-aminocyclopropane 1-carboxylate oxidase (ACO) converts ACC to ethylene, H_2_O, HCN, and CO_2_ in the presence of oxygen (Hamilton *et al.*, 1991; Ververidis and John, 1991; Bannenberg *et al.*, 2009) (Fig. 5). However, SAM homeostasis is directly linked to the plastid via the methionine synthesis pathway. Also, a putative plastidial SAM importer was found to bind Ca^2+^, indicating stress-adaptive regulation (Stael *et al.*, 2011). Plastidial *de novo* synthesis of methionine requires the formation of cystathionine from cysteine and phospho-homoserine by catalysis through cystathionine γ-synthase (CGS). Cystathionine β-lyase (CBL) then catalyses the conversion of cystathionine to homocysteine. Finally, methionine synthase (MS) methylates homocysteine, using 5-methyltetrahydrofolate as a methyl donor, into methionine (Hesse and Hoefgen, 2003), which is then exported to the cytosol. While there are three MS isoenzymes in Arabidopsis with AtMS3 localized in the plastid and AtMS1 and 2 localized in the cytosol (Hesse and Hoefgen, 2003; Ravanel *et al.*, 2004), CGS and CBL are exclusively localized in the plastid and were shown to be embedded in a complex regulatory network with other plastid localized biochemical pathways (Ravanel *et al.*, 1998; Watanabe *et al.*, 2021).

A wealth of data support the notion that SAM levels influence ethylene production in the cytoplasm, and the cytoplasmic Yang cycle seems to have a primary impact on the SAM levels and subsequent ethylene biosynthesis. Yang cycle-mediated MTA recycling is especially important for plants naturally producing a high ethylene level, like ripening tomato and rice, which is demonstrated by Yang cycle gene up-regulation. In Arabidopsis, Yang cycle genes are not ethylene dependent; however, presumably during increased demand for ET, elevated ethylene biosynthesis induces MTA recycling, in order not to inhibit ACS activity (for a recent review see Pattyn *et al.*, 2021). Despite the importance of cytoplasmic SAM and ACC levels for ethylene signaling, there are data that suggest at a direct role of the plastid in ethylene precursor production under certain conditions. In tomato, Barry *et al.* (2012) described a *lutescent 2* loss of function mutant that displays slower chloroplast development and altered fruit ripening compared with wild type. *Lutescent 2* was found to be the ortholog of the Arabidopsis *EGY1* gene, which encodes a plastid thylakoid membrane-localized zinc-dependent M50 type metalloprotease (Chen *et al.*, 2005). Similar to leaf tissue, also in tomato fruits the plastid development was impaired in the *lutescent 2* mutant indicated by reduced photosynthetic rates or by whitish fruits instead of green ones at the onset of fruit formation. Ethylene levels in the fruits of *lutescent 2* plants were reduced about 30% ­compared with the wild type, and application of exogenous ethylene was enough to alleviate the delayed onset of the ripening phenotype of *lutescent 2* fruits (Barry *et al.*, 2012). Interestingly, characterization of the *Orr*^*Ds*^ mutant, a dominant transposon-tagged tomato mutant deficient in the plastidial NADH dehydrogenase (Ndh) subunit NDH-M, revealed a delay in the onset of the fruit ripening phenotype as well as ~50% reduced ethylene levels emitted from fruits compared with the wild type (Nashilevitz *et al.*, 2010). Based on the observation that precursors of methionine biosynthesis are reduced by ~30% in the *Orr*^*DS*^ mutants, the authors concluded that the reduced ethylene levels are likely the result of reduced plastid-dependent ethylene production (Nashilevitz *et al.*, 2010). In summary, the data on plastid development-retarded mutants such as *egy1* in Arabidopsis (Guo *et al.*, 2008), *lutescent 2* (Barry *et al.*, 2012), and *Orr*^*Ds*^ (Nashilevitz *et al.*, 2010) show that in none of these mutants does the triple response for etiolated seedlings seem to be altered, and ethylene-caused phenotypes can be rescued by application of exogenous ethylene. This indicates that ethylene signaling seems to be functional and suggests that the ethylene-dependent phenotypes in these mutants are caused by reduced ethylene biosynthesis. A third indication for a role of the plastid in ethylene biosynthesis is that *CGS* transcript levels were found to be positively correlated with ethylene levels in ripening tomato fruits in order to sustain a high level of methionine biosynthesis (Katz *et al.*, 2006). However, in order to determine that indeed plastid-derived metabolites are the limiting factor in the ethylene biosynthesis pathway, the possibility of indirect regulation of the core ethylene biosynthesis pathway ACCs and ACOs has to be tested in these mutants. Therefore, given current state of knowledge, the direct signaling connection originating from the metabolic capacity of the chloroplast and ethylene signaling remains hypothetical.

### Indirect signaling connections

In contrast to the direct role of the chloroplast in ethylene signaling in a retrograde fashion, signaling via the ethylene responsive nuclear transcription factor ETHYLENE INSENSITIVE 3 (EIN3) has an anterograde function in chloroplast biogenesis itself. Liu *et al.* (2017) demonstrated that EIN3 interacts with PHYTOCHROME INTERACTING FACTOR3 (PIF3), a darkness-stabilized bHLH transcription factor, to repress the expression of most light harvesting complex (LHC) genes in the dark, thereby repressing expression of these genes and blocking the transition from etioplasts to chloroplasts. Upon exposure to light, phytochrome B-dependent degradation of EIN3 and PIF3 leads to *LHCA* and *LHCB* transcription, promoting chloroplast development (Liu *et al.*, 2017).

Plastids and mitochondria are also connected indirectly to ethylene signaling via second messenger-related mechanisms. The best described example for this is probably the mitochondrial unfolded protein response. In an elegant study using mutants and chemical treatments leading to impaired mitochondrial translation, Wang and Auwerx (2017) discovered that the mitochondrial unfolded protein response (UPR^mt^) leads to a transient ROS burst within the first 60 min of triggered stress in Arabidopsis. That correlated with an increased activating phosphorylation of MITOGEN ACTIVATED PROTEIN KINASE 6 (MAPK6) in the same time frame. MAPK6 activity led to an increase in transcription of *ACS6*, thereby activating the ethylene response leading to a part of the transcriptional changes observed for the initially elicited UPR^mt^, ultimately restoring mitochondrial protein homeostasis (Wang and Auwerx, 2017).

With respect to plastid protein translational stress caused by lincomycin treatment Gommers *et al.* (2021) report a substantial overlap in gene expression between ACC-treated plants and lincomycin-treated plants as well as in their respective phenotypes of cotyledon opening and hypocotyl length. Somewhat surprisingly, neither an increase in ethylene nor prolonged stabilization of EIN3 was reported after 3 d of lincomycin treatment, prompting the authors to hypothesize an ethylene-independent response for activating classical ethylene-dependent genes downstream of EIN3/EIL1. Genetic analysis excluded GUN1-dependent retrograde signaling for activation of the observed gene expression and cotyledon opening patterns. However, at this point it should be mentioned that Gommers *et al.* (2021) analysed ethylene and EIN3 levels on constitutively lincomycin-treated plants and the data presented in Wang and Auwerx (2017) suggest a short transient ethylene signal rather than a prolonged constitutive one. Therefore, it still cannot be excluded that the actual ethylene signal might arise earlier in the lincomycin-treated plants.

An interesting link between plastid-derived ROS and the ethylene response was observed in potato plants expressing cyanobacterial plastid-targeted flavodoxin (fld) (Arce *et al.*, 2022). In their meta-analysis of transcriptional profiles from fld-expressing Solanaceae, the authors detected common *cis*-elements in the promoter regions of genes involved in ethylene metabolism as a main target group of down-regulated transcripts due to fld expression and presumably reduced ROS levels in these plants (Arce *et al.*, 2022). Unfortunately, the study lacked resolution to draw further conclusions for plastid-derived ROS and ethylene signaling, but in our opinion it is an additional reason to further investigate the relation between these.

### Parallel signaling connection

This third category of plastid/mitochondria/ethylene signaling is intriguing because it requires both a functional ethylene response and functional organellar signaling but both of them are seemingly independent of each other in the first place. The Arabidopsis *ETHYLENE-DEPENDENT GRAVOTROPISM-DEFICIENT and YELLOW-GREEN1* (*EGY1*) gene encodes a thylakoid membrane-localized protease involved in chloroplast development in leaf mesophyll cells that was isolated as an ethylene-dependent gravitropism mutant (Chen *et al.*, 2005). Surprisingly it turned out that the *egy1* mutant displayed a wild type like triple response indicating that the ethylene signaling cascade is intact in these plants (Guo *et al.*, 2008). Through careful observation the authors noticed that *egy1* shows a severe delay in chloroplast development in general. With respect to hypocotyl gravitropism the authors observed that the number, size, and starch levels of endodermal plastids were reduced compared with the wild type. Through application of exogenous sucrose, starch fill levels of endodermal plastids were restored and in the presence of ethylene also the gravitropic response of the *egy1* mutant (Guo *et al.*, 2008). From these data we can conclude that in the case of ethylene-dependent hypocotyl gravitropism, ethylene signaling is necessary to keep cells in a state that allows them to bend, whereas the endodermal plastid allows the plant to sense the gravity vector in these hypocotyl cells.

## Salicylic acid

Of all the hormones addressed, SA is the least characterized. At present, indirect or complex mechanisms are still under investigation. Accumulation of SA has several layers of effects, triggering changes in expression of multiple genes, including nucleus-encoded proteins that are translocated to mitochondria and chloroplasts as well as through direct interaction with several proteins and biphasic accumulation of ROS in different subcellular compartments (Lu and Tsuda, 2020).

### Direct connection of organellar functions in salicylic acid synthesis

**Fig. 6.**
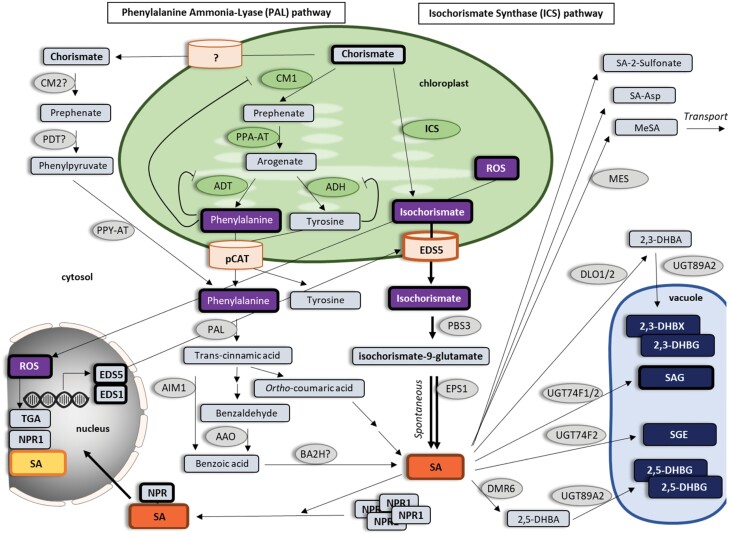
The role of organelles in salicylic acid signaling. Overview of salicylic acid (SA) synthesis via the isochorismate synthase (ICS) and phenylalanine ammonia-lyase (PAL) pathways starting from chorismate. ICS converts chorismate into isochorismate (IC) in plastids. EDS5 exports IC from the plastid into the cytosol where PBS3 converts it into isochorismoyl-9-glutamate and further into SA by EPS1. The PAL pathway converts chorismate into prephenate by CM1 or CM2. Prephenate is converted into arogenate by PPA-AT and further to tyrosine by ADH or phenylalanine by ADT. CM1, ADH, and ADT are negatively regulated by their corresponding amino acid products. Tyrosine and phenylalanine are transported into the cytosol where PPY-AT converts them to phenylalanine. Phenylalanine produced from both plastidal and cytosolic pathways is further converted into *trans*-cinnamic acid by PAL and then into SA via *ortho*-coumaric intermediate or benzaldehyde and benzoic acid. SA can be converted into functional or non-functional metabolites such as SA-2-sulfonate, SA-Asp and MeSA, or can be stored in the vacuole as SAG, SGE, 2,3-DHBX, 2,3-DHBG, 2,5-DHBX and 2,5-DHBG. Higher SA levels induce monomerization of NPR1, translocation into the nucleus and NPR1-dependent gene expression through direct interactions with TGA transcription factors. The genes explicitly mentioned in the text are highlighted (boxed with bold line). AAO, aldehyde oxidase 4; ADH, arogenate dehydrogenase; ADT, arogenate dehydratase; AIM1, abnormal inflorescence meristem1; Asp, aspartic acid; BA2H, benzoic acid 2-hydroxylase; CM, chorismate mutase 1; DHBA, dihydroxy-benzaic acid; DHBG, dihydroxybenzoic acid glucoside; DHBX, dihydroxybenzoic acid xyloside; DLO, DMR6-like oxygenase; DMR6, SA-5 hydrolase; EDS5, enhanced disease susceptibility 5; EPS1, enhanced Pseudomonas susceptibility 1; ICS, isochorismate synthase; MES, methylesterases; NPR1, Nonexpresser of PR gene 1; PAL, phenylalanine ammonia-lyase; PBS3, avrPphB susceptible3; PDT, prephenate dehydratase; PPA-AT, plant prephenate aminotransferases; PPY-AT, phenylpyruvate aminotransferase; SA, salicylic acid; SAG, SA 2-*O*-β-d-glucoside; SGE, salicylate glucose ester; UGT89A2, uridine diphosphate (UDP)-glucosyltransferase.

It is known that plants possess both an isochorismate synthase (ICS) and phenylalanine ammonia-lyase (PAL) pathway to synthesize SA, both starting from chorismate precursor (Zhang and Li, 2019) (Fig. 6). The ICS pathway is the primary route for SA production in Arabidopsis. However, the contribution of each pathway to the biosynthesis differs between plant species (Lefevere *et al.*, 2020). In the well-studied ICS pathway, isochorismate is produced from chorismate in chloroplasts and transported to the cytosol, where SA is synthesized (Rekhter *et al.*, 2019). Compartmentalization of the proteins responsible for the synthesis and transport control unidirectional forward flux to protect the pathway against evolutionary forces and pathogen perturbations (Rekhter *et al.*, 2019). On the other hand, it is unclear whether the steps of the PAL pathway leading to the SA precursor phenylalanine take place in the chloroplast, cytosol, or both simultaneously (Lefevere *et al.*, 2020). However, in the next steps of the PAL pathway, cytosol and peroxisomes are involved in SA synthesis (Murphy *et al.*, 2020). Once synthesized, SA may undergo a number of chemical modifications including glucosylation, methylation, sulfonation, hydroxylation, and amino acid conjugation (D’Maris Amick *et al.*, 2011). Some modifications inactivate SA in its regulatory roles to fine-tune SA activity, whereas others serve as temporary pools for its storage, such as SA glucoside, a vacuole-localized SA reserve (Ding and Ding, 2020).

### Direct signaling connections: multiple regulatory feedback loops linking chloroplasts and SA signaling

SA signaling is known to be tightly interconnected with ROS signaling, which is also involved in several stress responses including pathogen attack (Bleau and Spoel, 2021). The transient phase of ROS accumulation occurs within minutes after infection and is mostly apoplastic and tightly linked to the activities of RBOHs, plasma membrane NADPH oxidases (Kadota *et al.*, 2014; Li *et al.*, 2014). In potato, RBOHD and SA regulate the immune response through a complex regulatory feedback loop, as RBOHD is required for the spatial accumulation of SA, and conversely, RBOHD is under the transcriptional regulation of SA (Lukan *et al.*, 2020). RBOHD-dependent ROS production is also regulated by SA in Arabidopsis (Liu and He, 2016). The second, more sustained phase of ROS accumulation occurs in different compartments, including the apoplast, chloroplasts, mitochondria, and peroxisomes (Shapiguzov *et al.*, 2012), and is associated with the establishment of defense responses and signaling for and/or execution of hypersensitive response cell death in incompatible interactions (Lu and Yao, 2018). Whether SA plays a role in this signaling has not been fully deciphered yet. Assumptions that SA signaling is not involved in cell death execution (Ochsenbein *et al.*, 2006; Yao and Greenberg, 2006; Zurbriggen *et al.*, 2009) were later questioned by Straus *et al.* (2010). They suggested that chloroplastic ROS acts as a flexible spatiotemporal integration point leading to opposite SA signaling reactions in infected and surrounding tissue to control the propagation of cell death. Interestingly, in potato hypersensitive response, ROS generated in the chloroplasts around the cell death zone are involved in two different SA-dependent and SA-independent plant response processes, which are spatially regulated. Strongly oxidized disordered chloroplasts in the cells on the edge of the cell death zone play a role in SA-independent hypersensitive response programmed cell death signaling, while the cells with moderately oxidized chloroplasts farther away from the cell death zone evidence SA-dependent signal transmission to neighboring tissue at the transcriptional level (Lukan *et al.*, 2020). Chloroplastic ROS is involved in the up-regulation of genes upstream of SA accumulation through retrograde signaling, such as *ENHANCED DISEASE SUSCEPTIBILITY1* (*EDS1*) (Ochsenbein *et al.*, 2006) and *ENHANCED DISEASE SUSCEPTIBILITY5* (*EDS5*) (Nomura *et al.*, 2012).

### Indirect mechanisms: SA binds to proteins from different subcellular compartments

Recently, multiple SA-binding proteins (SABPs) with different structures, localizations, and functions have been identified (Pokotylo *et al.*, 2019). However, a direct function in plant physiology is currently not known for them all. Slaymaker *et al.* (2002) identified SALICYLIC ACID-BINDING PROTEIN 3 (SABP3) as a chloroplast β-carbonic anhydrase, which exhibits antioxidant activity in the soluble fraction of purified tobacco leaf chloroplasts. They proposed it has role in plant defense, perhaps through antioxidant activity (Slaymaker *et al.*, 2002; DiMario *et al.*, 2017). Treatment with SA altered the localization of the protein, which entered the cytoplasm, where interaction with SA and NONEXPRESSOR OF PATHOGENESIS-RELATED GENES 1 (NPR1) occurs (Medina-Puche *et al.*, 2017). Another protein with antioxidative activity in plant immunity and identified as a SABP is glutathione *S*-transferase (GSTF) (Tian *et al.*, 2012) with roles in the metabolism of antimicrobial compounds and detoxification of mycotoxins (Gullner *et al.*, 2018). Dixon *et al.* (2009) characterized AtGSTF2 and AtGSTF8 as chloroplastic proteins and AtGSTF10 as vacuolar. SA binding inhibits GSTF activity, which modulates glutathione homeostasis and thus the cell redox state (Nazar *et al.*, 2017). Another redox-related protein that binds SA is chloroplastic TRX-m1 (Manohar *et al.*, 2015). The effect of SA binding on TRX-m1 activity has not yet been established; however, the activity of cytosolic TRX-h3 and TRX-h5 is required for NPR1 monomerization (Tada *et al.*, 2008). Other chloroplastic proteins that bind SA are glyceraldehyde 3-phosphate dehydrogenase (GAPDH) isoforms AtGAPA-1 and AtGAPA-2, which play a role in the Calvin cycle (Pokotylo *et al.*, 2019). In humans, SA binding suppresses GAPDH translocation to the nucleus, which plays a role in the regulation of replication of some viruses (Choi *et al.*, 2015), while the role of this interaction in plants has not yet been deciphered. Another interaction with a potential role in plant antivirus defenses is suppression of mitochondrial α-ketoglutarate dehydrogenase (KGDE2) by SA in tomato, which occurs through direct binding (Liao *et al.*, 2015). In contrast to viral resistance, the results of a recent study in tomato showed that KGDE2 plays a negative role in plant basal defense against *Pseudomonas syringae* in association with the SA defense pathway (Ma *et al.*, 2020). Whether this occurs through direct binding needs to be elucidated. The presence of SABPs exhibiting a wide range of affinities for SA, combined with the varying SA levels found in specific subcellular compartments, in different tissues, at different developmental stages, or during responses to environmental cues, provides tremendous flexibility and multiple mechanisms through which SA effects can be utilized in plants.

## Outlook

From the data published up to now, it can be concluded that phytohormone and organellar signaling works in concert at various levels. However, the studies discussed in this review also make clear that the connections between organellar and phytohormone signaling depend on complex factors such as tissue type, developmental status, and environmental stress conditions (Aerts *et al.*, 2021; Cackett *et al.*, 2022). A fact that further makes comparisons between studies difficult is that often, although arising from the same signaling pathway, different components are monitored. Subsequently, from those singular datasets conclusions for the whole pathway are implied. However, with the wealth of knowledge we currently have on processes like phytohormone biosynthesis, perception, and signaling as well as their interconnections to other signaling pathways at various points and levels (Ludwików *et al.*, 2014; Blázquez *et al.*, 2020; Binder, 2020; Depaepe *et al.*, 2021; Müller, 2021; Müller and Munné-Bosch, 2021; Cackett *et al.*, 2022), it becomes clear that taking single elements of these pathways does not generally permit predictions for the whole interconnected signaling process. These facts are increasingly being taken into consideration in current research and they will provide a more holistic framework for how organelles such as mitochondria and plastids are connected to the core phytohormone responses. Linking hormonal signaling to intracellular organellar communication brings new perspectives to and better understanding of plant signaling networks. In particular, it shows how developmental processes and responses to stress can be modulated through the functional state of organelles. Taking this perspective into account, future experiments can be designed to capture direct and indirect connections at the metabolic, transcriptional, and physiological level. In a long-term perspective, this knowledge will guide future functional studies and lead to improved crop breeding strategies for stress resilience.
